# Policy considerations to achieve practical ethics: closing the gap between ethical theory and practic

**DOI:** 10.18502/jmehm.v13i8.4075

**Published:** 2020-08-25

**Authors:** Mansure Madani, Nazafarin Ghasemzadeh, Ali Dizani, Ahad Faramarz Gharamaleki, Bagher Larijani

**Affiliations:** 1 *PhD Candidate in Medical Ethics, Medical Ethics and History of Medicine Research Center, Tehran University of Medical Sciences, Tehran, Iran;* *PhD Candidate in Medical Ethics, Medical Ethics and History of Medicine Research Center, Tehran University of Medical Sciences, Tehran, Iran; Department of Medical Ethics, School of Medicine, Tehran University of Medical Sciences, Tehran, Iran. *; 2 *Researcher, Department of Medical Ethics, Faculty of Medicine, Urmia University of Medical Sciences, Urmia, Iran. *; 3 *Researcher, Qom Seminary and Department of Islamic Knowledge and Humanities, Amirkabir University of Technology, Tehran, Iran. *; 4 *Professor, Department of Islamic Theology and Philosophy, University of Tehran, Tehran, Iran.*; 5 *Professor, Endocrinology and Metabolism Research Center, Endocrinology and Metabolism Clinical Sciences Institute, Tehran University of Medical Sciences, Tehran, Iran.*

**Keywords:** Intrinsic and extrinsic coercion, Ethics and law interaction, Policies and intrinsic motivation, Social ethics.

## Abstract

Social and professional behaviors are driven by extrinsic as well as intrinsic factors including executive rules and regulations enacted by extrinsic agents through coercion, police force and penalties. Despite their effectiveness, these mechanisms undermine the fact that ethics is an intrinsic human quality. The present study seeks strategies to apply extrinsic coercion as an incentive to direct ethics as an intrinsic value.

Ethical behaviors driven by intrinsic motivations are more permanent and less costly. Legal force can either strengthen or weaken intrinsic requirements. Extrinsic conditions such as considering the interests, attitudes and preferences of others, involving people in the regulation and execution of law, justification of law, avoiding excessive punishment or rewards, and indirect support of ethics by establishing the appropriate social context can help boost intrinsic requirements in individuals.

Ethics will not be practically established unless we harness individuals’ ‘willingness to act’ as an essential determinant for ethical behavior. This requires adoption of a more psychological approach to ethics. If this aspect of ethical behavior is considered in regulations and executive processes, extrinsic forces can strengthen intrinsic requirements and spread ethics.

## Introduction

Voluntary behaviors occur when an individual is willing to do something. Any factor, whether intrinsic or extrinsic, that affects individual willingness to do something can lead to a certain behavior. 

Intrinsic factors are numerous, and sometimes inconsistent, factors that drive our desires from within. They are basic needs that make an individual perform an action to gain pleasure or relieve pain. These basic needs are not negligible and play a significant role in forming our decisions and behaviors. Later on, higher-order needs such as eagerness to know, aestheticism and humanitarianism inspire human actions (1). Nevertheless, not all these factors lead to action and some may even contradict each other. It is our values and preferences that provide the motivation for actions (2). Ethics is an intrinsic factor that controls social behavior and is guaranteed by individual conscience. We accept ethics when we prioritize moral behavior over our values and desires. Some of these values are imposed by others and are internalized and personalized.

Effects of intrinsic factors and desires on human behavior have been mostly neglected in the literature. In the 18^th^ century, the correlation between intrinsic factors such as emotion and character and human behavior was studied and interventive measures in philosophy (3), psychology (4), professional ethics (5, 6) and medical ethics (7) were seriously initiated. 

Extrinsic factors include law enforced by the police force and penalties, as well as customs imposed through social pressures such as reprimand and humiliation. These factors may be internalized and turn into individual values to direct human actions. On the other hand, the individual may inwardly reject them but feel obliged to accept them out of fear or due to external coercion (1).

**Figure 1:**
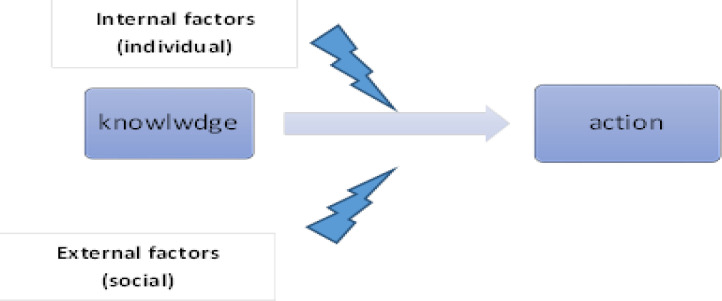
Turning theory to practice (law and custom as extrinsic factors, and ethics as an intrinsic factor)

Law enforcement is a mechanism to control individual, professional and organizational behavior. Executive laws and regulations use rewards or punishment to control human behavior and establish social order. It is a powerful means with a relatively predictable control outcome. Enforcement of law is an essential aspect of human relations because justice cannot be established solely by common sense and human accountability (8). However, despite its high costs, law alone is insufficient in controlling all behaviors (9). Thus, two sets of intrinsic and extrinsic factors determine human behavior, as illustrated in figure 1.

Intrinsic motivation is strong and persistent and needs no external force for action, but extrinsic motivation is temporary and weak. A law is more likely to be ignored when people abide by it out of fear without really believing in it. Any sort of behavior that is controlled by such external forces may be performed in secret and may even return to its earlier state when the force in action subsides, for example when there is no policeman or the law is abandoned (1, 10). Therefore, it is suggested that strong intrinsic motivations be employed to control human behaviors, and other costly/inefficient mechanisms of law be reserved for more risky cases. Moreover, law and executive processes should be redesigned to reinforce intrinsic motivation and inflict the least possible damages.

One significant and unpleasant detail that is often neglected in controlling behavior is that extrinsic intervention programs (punishment or reward) seriously damage or eradicate intrinsic motivation. The individual will gradually substitute intrinsic motivation with an extrinsic factor and violate law when there is no police officer or human observer (1,10, 11). In other words, when law intervenes, a persistent and strong motivation turns into a temporary and inadequate obligation.

There is ample psychological proof for this issue. For instance, children who receive prizes for studying will stop it when they are not rewarded anymore. Those who are interested in studying will do it for a prize (free pizza) rather than their own interest. This implies that rewarding has in some cases reverse effects and weakens intrinsic desire for an action, such that the individual abandons the action when he feels he will not be rewarded (1). This is also true for big punishments. Small punishments may change a person’s attitude, but big ones, though more effective in changing behavior, have little impact on individual willingness to do something (1,11,12). Psychologists have proposed some theories, including the cognitive dissonance theory, attribution theory, and self-justification theory to explain this issue. They briefly note that people generally tend to justify their actions by reasons that seem logical to them. They carry a self-conception that is always positive and gives them a sense of self-esteem. Therefore, they try to do things that are consistent with their self-conception (I am a respected person and never do anything to hurt my reputation). Otherwise, they will be distressed and anxious (1, 13). Moreover, people process and associate information to be consistent with each other. That is to say, a new perception needs to be consistent with earlier ones and not hurt one’s self-esteem (13). Excessive reward or punishment systems make individuals adapt themselves to those extrinsic factors (I will do something to be rewarded), and once the system is no longer in place, they will feel less obliged to do the same thing. But when the reward or punishment system is not excessive, individuals will attribute their following social norms to their own willpower rather than external factors, and will feel bound to the law even if there is no observer (I am not so cheap to be by inspired by such an incentive; I like it and I do it) (1, 12). Similarly, it has been shown that people are less likely to follow rules when the regulatory system is weak compared to when there is no system (14). This psychological evidence explains why attitudes need to be internalized. 

However, external interventions are not all negative. Studies show that laws and rules help internalize positive behaviors and reinforce ethics under certain circumstances. When the logic behind rules is explained, justified, taught and enculturated, self-control is strengthened among people and they automatically perform the action, even when the external force is removed (12).

Ethical behaviors will be driven by intrinsic incentives when people are appropriately informed about the logics behind ethics, or are actively involved in the process of providing ethical training (15). Once ethics is internalized, extrinsic forces can be applied with care to guide and reinforce intrinsic incentives but not turn them into external obligations. 

The present study focuses on social and professional ethics. Ethical behaviors are formed by intrinsic incentives and willingness to do things. Therefore, the present study seeks to find mechanisms to modify laws and executive processes to lead to ethical behaviors. It examines the effects of extrinsic coercion on ethical behaviors and the way laws and executive processes can be designed to trigger intrinsic incentives. To our knowledge, this has not been carefully examined in the literature and is occasionally referred to in social psychology. In this study, we intend to discuss the issue and offer some solutions. Here, law refers to any obligation with executive guarantee/control, including regulations, processes, etc.

## Method

This is an interdisciplinary and analytic study. The following keywords were first explored in valid databases in psychology, sociology, and ethics: ethical knowledge, ethical practice, ethical weakness, motivation, willpower, and awareness. The resulting essays were filtered and irrelevant ones were removed. The remaining essays were carefully studied and the authors’ viewpoints were extracted. Related books on the topic were used in the course of our analysis, and considering the interdisciplinary nature of the study, reference books were also used to increase the validity of the study.

 When the primary search and analysis was done, new keywords were obtained, including internalizing ethics, moral development or ethical growth, socialization, structure and agency, intrinsic and extrinsic incentives, psychology of policy-making, and the unconscious. These keywords were further used to achieve better search results across different fields.

## Results

**The interaction between law and ethics and its effects on ethical behavior**

The relationship between ethics and law can be investigated from different aspects:

***1. Legal Enforcement of Ethics***

The simplest way to guarantee ethics is to legally enforce ethical behavior by setting rewards or punishment in connection with any ethical rule. This is accompanied by some problems, both in theory and in practice. Laws essentially restrict individual freedom and have to be minimized. It is sometimes unethical for law to intervene in private aspects of human life and make regulations for humanitarian and self-sacrificing acts. In addition, laws are administered by force, which can be abused and should therefore be limited to certain cases. On the other hand, they are costly and it is difficult in many cases to provide lawful basis, supervisory administration and punishment for ethical concerns such as telling lies (9).

Laws are essentially inefficient in the field of human emotions such as abhorrence and jealousy, and can only cover a limited number of ethical issues. Intrinsic incentives such as emotions and sentiments often instigate actions that cannot be regulated and modified by reward or punishment.

***2. Ethical Audit of Law***

Laws can be regulated and audited based on ethical considerations. In fact, laws attract individual compliance and obedience because they are supported by ethics; also, the law claims legitimacy and morality of its orders (9). However, even an ethically-passed law may lead to unethical problems when enforced in practice. For instance, documentation in nursing practice is only a recording of unusual events, wearing out the time and energy of healthcare providers. Thus, ethical audit is necessary but not sufficient on its own. 

***3. Creating an Ethical Atmosphere/ Climate***

 It is very important and valuable to raise ethical humans, but sociopsychological studies show that it would be nearly impossible unless we create the infrastructural requirements and background (1). In other words, we can use laws and regulations to reform social structures and provide an atmosphere in which unethical people are compelled to act ethically. The opposite is also true, i.e., ethics is fostered and facilitated when the costs of unethical behavior rise. Laws and executive processes can provide a framework for social interactions through effective management of political decision-making, conducting appropriate educational programs, and establishing positive incentives such as rewards for voluntary community services, in order to persuade unethical people to adhere to ethics. Laws can also engage people in self-sacrificing ethical acts like helping the needy. These are voluntary activities and cannot be legally enforced, but can be ethically organized. Laws can indirectly support such charitable acts by making them tax- free (9). 

***4. Contribution of Law to Self-Control***

Laws and processes can significantly contribute to establishing ethical habits and characters. One key cause of unethical action is that the individual feels unwilling to do something but cannot help himself. Disciplinary rules enhance self-control and self-regulation, and help establish and internalize ethics (12). However, we should bear in mind that these interventions are temporary and only aim to internalize ethics. Extrinsic factors, such as laws that are intended to establish ethical habits, are most effective when coupled with training, enculturating and convincing the target population. Fastening seatbelts is an example of how enculturating and coercion worked together to make drivers abide by the law. In this regard, excessive reward or punishment systems should also be avoided.

***5. Policies that Maintain or Reinforce Intrinsic Incentives***

Another key aspect in the interaction of ethics and laws, which is mostly neglected, is to pass laws and executive processes that reinforce or at least do not harm internal and ethical motivations. This is a complicated issue and policy-makers can make better decisions if they become more informed about harmful and useful policy-making capabilities and solutions. For example, people rarely agree to obey rules that are against their religious beliefs (16).

The present study adopts a psychological approach and offers a host of effective mechanisms based on the distinction between internal and external obligation. Below, policies with the least effects on intrinsic incentives are examined.

*A. Passing laws with highest compatibility with individual attitudes and values:* Laws need to be at least partially in accordance with individual attitudes and ethics to be accepted and effective. Otherwise, they will cause conflicts, biases, ambivalence and pangs of conscience in people and will be rejected, which will finally harm intrinsic motivations (16). 

*B. Serving and supplying the interests of the majority of people:* Most people work for their interests and do whatever they can to maximize their personal profit or benefit. An efficient legal system supplies the interests of the majority and allows even profiteers who have little ethical concerns to achieve what they like. By doing so, it strengthens intrinsic motivations. If agents of a party (physicians, nurses, etc.) partake in regulating meetings, their interests might be represented. However, it is a relative objective and may not be realized in all cases (9) because sometimes individual and social interests are in conflict. In such cases, the law should be justified and people should be convinced to believe that abidance by law brings them more benefit.

*C. Justification of law:* Laws need to be ethically justified to be valid. It is essential to convince people of the significance of having a legal system that safeguards community life and serves the benefits of the majority (17). Justification of law and enculturating are necessary for ensuring that laws comply with social interests, which is realized when policy-makers convince people and attract their cooperation. This will decrease cognitive dissonance, which will otherwise give rise to social and individual conflicts and lead to anxiety, ambivalence and bias. In fact, justification of law aims at boosting intrinsic incentives and reducing extrinsic coercion. For example, some physicians never obtain patients’ informed consent because they think it is not required or even against patients’ well-being. If they are convinced of its effectiveness for treatment, they will surely feel more inclined to cooperate (9, 16).

*D. Involvement in policy-making:* Involving people in policy-making activities (such as formulating, approving or enforcing the law) contributes significantly to inspiring intrinsic incentives. The more people are engaged in these activities, the more likely it will be to achieve practical ethics and implement laws.

When people are involved in an activity, they feel motivated and try their best to show greater levels of commitment. In group or participatory activities, people are involved both mentally and physically, feel more attached to the group and work towards collective objectives. Internalization of ethics occurs when people are given a chance and a role in the group and feel qualified in contributing to the community. Cooperation has been proved to enhance self-esteem, self-efficacy and self-sufficiency, and makes the individual feel motivated to freely engage in group work (18-20). It also helps people be more considerate and tolerant towards others and their rights because they will be more inclined to understand them (21). Studies show that cooperation improves learning and modifies radical attitudes (22-24). 

A cooperative atmosphere affects incentives and helps decision-makers gain an insight into the reality of the society and make realistic decisions (25) while delivering more skillful and empowered individuals (26). In such an atmosphere, cognitive assonance will also increase. As mentioned earlier, people always try to justify their behavior and tend to associate different perceptions that form their attitudes. When individuals participate in making regulations, they can hardly justify any act of their own that violates the law because doing so would be in contrast with their sense of self-esteem.

Cooperation is achieved by vote, survey or similar means. In an organization, for example, staff representatives participate in decision-makings (19). Delegation of authority to lower ranks in an organization is another form of cooperation that makes the staff feel worthy and perform their duties better (27-29). Other types of organizational cooperation include forming working committees, educating managers and supervisors, identifying and engaging qualified staff in organizational affairs, and persuading the staff to express their opinions and come up with new ideas (30). Lack of formal and informal mechanisms to get feedback from the system makes the staff feel detached from their managers and stay silent. When they feel their voices are not heard by their top managers, they feel indifferent towards organizational goals (30). This silence will drive the organization away from its planned objectives and will provide little information for managers to intervene and make the required reformations (31, 32). 

*E. Avoiding excessive reward or punishment systems: *Big rewards and demanding or strict rules may control behaviors but will kill intrinsic motivation and behaviors will be driven by extrinsic incentives. There is enough evidence for this fact: children who are punished slightly for playing with a toy are less likely to play with it in secret (1). This has been explained in the introduction section. 

*F. Facilitating social solidarity:* Any community can provide the setting for social solidarity and foster ethical virtues. It can also support ethical behavior by appropriate education, persuading desirable social activities, and rewarding voluntary activities (9). Social systems have been shown to be highly influential in creating an environment that boosts intrinsic incentives. Common ethical values rely on social solidarity and a sense of commitment and belonging to the group. Laws and executive processes can create an appropriate framework for social cooperation and solidarity and contribute to ethics (33). Laws can organize certain institutes such as non-governmental organizations (NGOs) and professional associations that strengthen solidarity. People voluntarily join such institutes and, once they are in, follow the rules. 

These groups are democratic and non-government-backed. Therefore, individual factors such as fear of press disclosure of news make people control their behavior. NGOs have multiple channels to professional ethics. They support ethics in society, associations and professions, and their existence relies heavily on adhering to ethics (34).

Psychological studies confirm internalization of group rules. People tend to identify with and follow group disciplines. They comply with group standards and imitate others’ behaviors, and internalize collective norms as their own beliefs. They also perform their pre-defined roles and are ready to pay any costs to preserve the group. This implies that institutional norms are powerful entities that shape and direct individual desires (1). Professional ethics will be further strengthened if NOGs become more active and participate in administrating their own affairs.

## Conclusion

**Figure 2:**
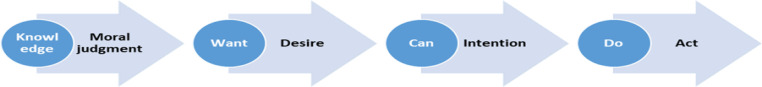
Turning theory to practice

The gap between ethical theory and practice is a problem in many aspects of social sciences. It is yet to be found why people do something against their own beliefs. A host of schools in psychology and philosophy have considered the process of turning theory into practice and have provided a medium to intervene and reduce this gap. The two most important mediators are will and intention. Knowledge and awareness will end in action when individuals have enough motivation and willingness to do it. Moreover, they should have the ability to do the action and obstacles should not be so big that they feel demotivated. Figure 2 shows the process of turning theory to practice.

Ethical judgment is acquired by direct or indirect education, which will end in action if coupled with individual willingness. Therefore, laws and executive processes can help actualize practical ethics in two ways: strengthening/weakening intrinsic incentives, increasing/decreasing practical obstacles.

Laws are essential extrinsic forces and have nothing to do with intrinsic incentives. However, studies indicate that they mutually affect each other. External obligations can either strengthen or suppress intrinsic incentives. However, they should be applied with caution because laws have potential drawbacks and human emotions are significant factors in performing ethical acts. Considering individual attitudes and interests, justifying and enculturating laws, promoting cooperation, appropriate use of reward and punishment, and facilitating social solidarity are mechanisms that help boost ethics.

**Figure 3:**
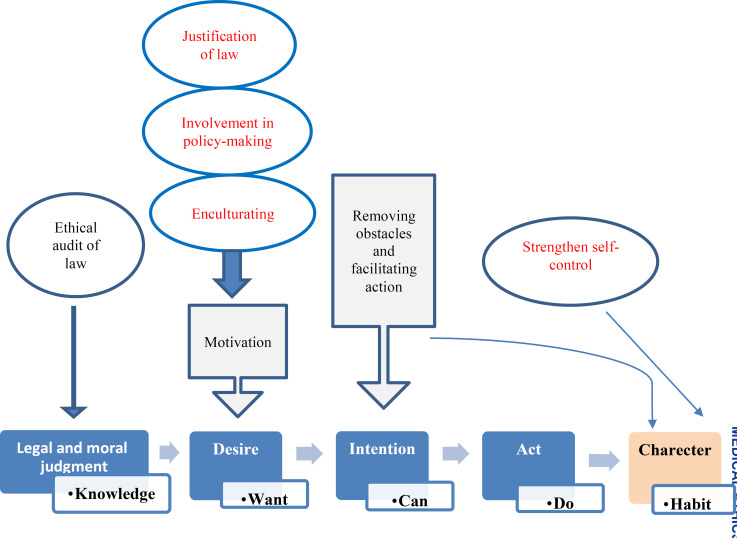
The process of turning theory into practice

Creating an ethical atmosphere facilitates ethical behaviors and lowers the costs of ethical behavior while increasing the costs of unethical actions. In fact, people realize that doing ethical acts is more beneficial. Correct laws and executive processes can create such an atmosphere and ethical auditing can gradually remove potential obstacles. This is mostly achieved by providing the required infrastructures to develop professional associations and NGOs. These institutes are run by people and have great roles in promoting ethics. They also facilitate ethical and even self-sacrificing behaviors. Passing laws and applying coercion can help turn them into routine ethical behaviors, which are later internalized by training and enculturating. Figure 3 shows the effects of policies on the gap between theory and practice.

Laws are imposed on people by force and make them avoid wrong doing, but ethics can cause people to voluntarily do the right thing. Regulators are urged to avoid passing laws that damage intrinsic incentives. Instead, they should, pass laws that strengthen intrinsic incentives to decrease the likelihood of violating laws and increase voluntary abidance by rules even in the absence of laws. In promoting ethics, policy-makers can benefit from psychological insights to achieve effective rules and laws.

## Notes:

**Citation to this article:**

Madani M, Ghasemzadeh N, Dizani A, Gharamaleki AF, Larijani B. Policy considerations to achieve practical ethics: closing the gap between ethical theory and practice. J Med Ethics Hist Med. 2020; 13: 8.
